# Metabolomics of Major Depressive Disorder: A Systematic Review of Clinical Studies

**DOI:** 10.7759/cureus.23009

**Published:** 2022-03-09

**Authors:** Livia N F. Guerreiro Costa, Beatriz A Carneiro, Gustavo S Alves, Daniel H Lins Silva, Daniela Faria Guimaraes, Lucca S Souza, Igor D Bandeira, Graziele Beanes, Angela Miranda Scippa, Lucas C Quarantini

**Affiliations:** 1 Medicine, Laboratório de Neuropsicofarmacologia, Serviço de Psiquiatria do Hospital Universitário Professor Edgard Santos, Universidade Federal da Bahia, Salvador, BRA; 2 Medicine, Programa de Pós-Graduação em Medicina e Saúde, Faculdade de Medicina da Bahia, Universidade Federal da Bahia, Salvador, BRA; 3 Medicine, Laboratório de Neuropsicofarmacologia, Serviço de Psiquiatria do Hospital Universitário Professor Edgard Santos, Universidade Federal da Bahia, Salvador, Brazil, Salvador, BRA; 4 Medicine, Faculdade de Medicina da Bahia, Universidade Federal da Bahia, Salvador, BRA; 5 Medicine, Departamento de Neurociências e Saúde Mental, Faculdade de Medicina da Bahia, Universidade Federal da Bahia, Salvador, BRA

**Keywords:** human studies, biomarker, metabolomic, depression, major depressive disorder

## Abstract

Although the understanding of the pathophysiology of major depressive disorder (MDD) has advanced greatly, this has not been translated into improved outcomes. To date, no biomarkers have been identified for the diagnosis, prognosis, and therapeutic management of MDD. Thus, we aim to review the biomarkers that are differentially expressed in MDD. A systematic review was conducted in January 2022 in the PubMed/MEDLINE, Scopus, Embase, PsycINFO, and Gale Academic OneFile databases for clinical studies published from January 2001 onward using the following terms: “Depression” OR “Depressive disorder” AND “Metabolomic.” Multiple metabolites were found at altered levels in MDD, demonstrating the involvement of cellular signaling metabolites, components of the cell membrane, neurotransmitters, inflammatory and immunological mediators, hormone activators and precursors, and sleep controllers. Kynurenine and acylcarnitine were identified as consistent with depression and response to treatment. The most consistent evidence found was regarding kynurenine and acylcarnitine. Although the data obtained allow us to identify how metabolic pathways are affected in MDD, there is still not enough evidence to propose changes to current diagnostic and therapeutic actions. Some limitations are the heterogeneity of studies on metabolites, methods for detection, analyzed body fluids, and treatments used. The experiments contemplated in the review identified increased or reduced levels of metabolites, but not necessarily increased or reduced the activity of the associated pathways. The information acquired through metabolomic analyses does not specify whether the changes identified in the metabolites are a cause or a consequence of the pathology.

## Introduction and background

Major depressive disorder (MDD), one of the most common psychiatric conditions, has a major impact on health systems around the world, with a worldwide prevalence of 17% and an annual incidence of around 6% in the general population [1]. It is associated with an increased risk of cardiovascular disease, metabolic syndrome, obesity, stroke and increased global mortality correlated with metabolic changes [2].

However, biomarkers have not yet been identified for the diagnosis, prognosis and therapeutic management of MDD. Such biomarkers could be the metabolites of cells, tissues and body fluids, such as peptides, amino acids, saccharides, phospholipids, coenzymes and nucleotides [3]. The investigation of molecules through metabolomic analysis could assist in the discovery of biomarkers potentially related to the predisposition, development and prognosis of MDD and other mental illnesses [4].

The possible benefits to psychiatry range from a better understanding of the pathophysiology of MDD [5,6] to novel strategies for its management, such as tests that suggest the best treatment option for patients according to their metabolomic profile, or tests that are capable of monitoring the metabolic-specific response to treatment. Additionally, such tests might even improve the staging of MDD and reveal personal predispositions for the condition.

The current work provides a systematic review of clinical studies of metabolomics in MDD. We aimed to identify the main metabolites altered in the bodily fluids of patients with MDD, whether under treatment or not.

## Review

Methods

A systematic review of the literature was conducted, following the Preferred Reporting Items for Systematic Reviews and Meta-Analyses (PRISMA) guidelines [7] and the recommendations of the Cochrane Handbook for Systematic Reviews of Interventions [8], where applicable, and was registered in the International Prospective Register of Systematic Reviews (PROSPERO) database (CRD42020205879).

A clinical question was defined: “Which metabolites are altered in patients with active depression, whether under treatment or not?” This question guided the eligibility criteria and the literature search in the databases.

Eligibility criteria

The criteria for inclusion of studies were as follows: original studies published from 2001 onward evaluating metabolites in the bodily fluids of subjects with a clinical diagnosis of MDD under treatment or not; aged between 18 and 71 years old and no diagnosis of bipolar depression or other psychiatric disorders. Due to the possibility of influencing metabolomic analysis, we excluded studies that evaluated patients under phytotherapic interventions, with comorbidities that may interfere with the metabolic analysis such as viral hepatitis and acquired immunodeficiency syndrome, or women in the pregnancy-puerperal cycle. Studies that did not report the instrument used for the assessment of depression were also excluded. Case reports, reviews, editorials, letters, poster abstracts, and guidelines were excluded. There were no language restrictions.

Literature search

The literature search was performed in January 2022. The last literature search was performed on January 31, 2022. For PubMed/MEDLINE, Scopus, Embase and Gale Academic OneFile, the following terms were used: “Depression” OR “Depressive disorder” AND “Metabolomic.” For PsycINFO, studies were filtered for those conducted only in humans, and a different search strategy was used: (“Major depression” OR “Dysthymic disorder” OR “Endogenous depression” OR “Late life depression” OR “Recurrent depression” OR “Treatment resistant depression” OR “Depression” OR “Depressive disorder” OR “Major depressive disorder”) AND “Metabolomic.”

Study selection and data extraction

Duplicates were eliminated before selection. Afterward, the citations were independently screened by four reviewers (LG, BC, GA, LS) in terms of titles and abstracts. The four reviewers then independently assessed the full text of selected studies. For each study, we extracted the following in a standardized spreadsheet: i) first author and year of publication; ii) sample and treatment (if any); iii) instrument used for diagnosis and stratification of depression; iv) analyzed bodily fluid; v) method of metabolomic analysis; vi) differences in the metabolic profiles of patients with MDD and controls.

Risk of bias assessment

The quality of the included studies was evaluated by two reviewers (DF, DL) using the Strengthening the Reporting of Observational Studies in Epidemiology (STROBE) criteria [9]. Discrepancies between the two reviewers were resolved by consensus.

Results

**Figure 1:**
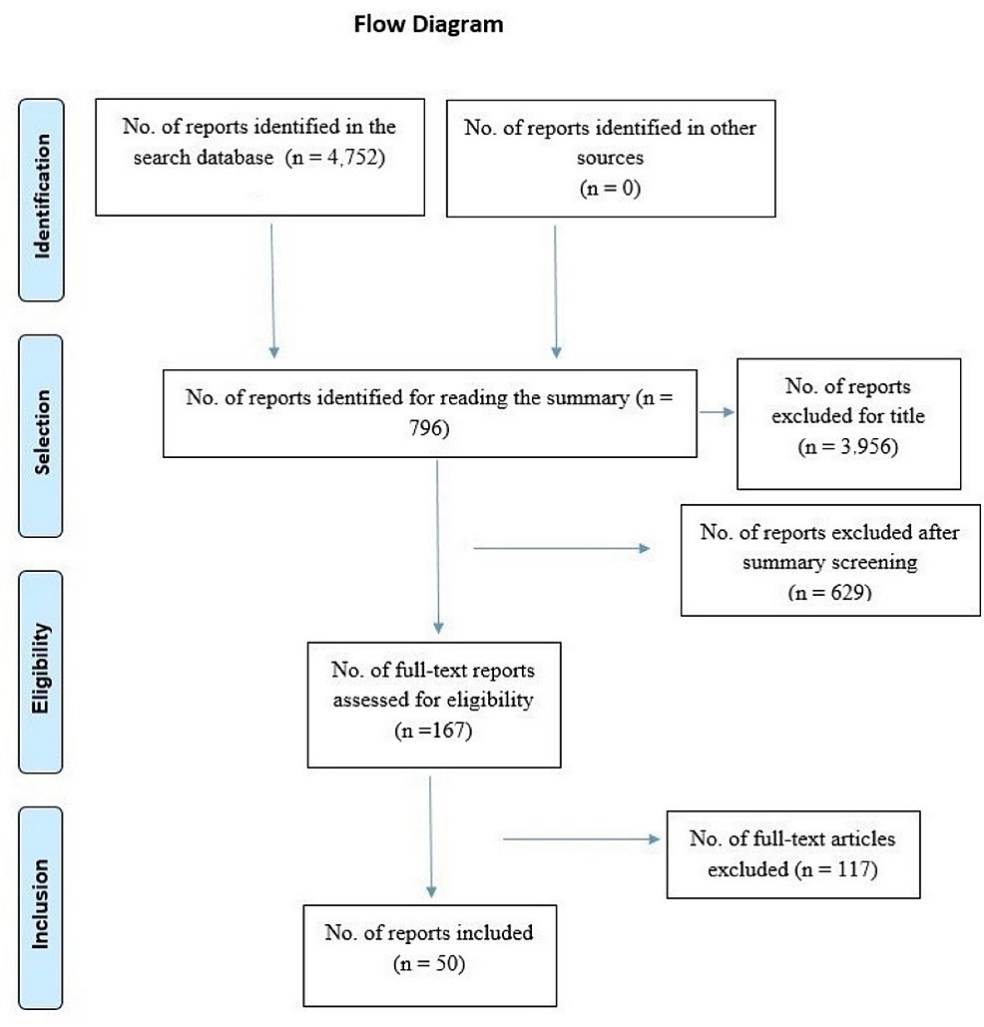
PRISMA diagram

During the initial search of databases, 4,752 articles were found. After reviewing the titles and abstracts, 167 were selected for full reading and 50 were included for analysis and data extraction (Figure 1).

Of the included studies, 19 were conducted in China, 14 in the United States, seven in Japan, two in Finland, two in the United Kingdom, and one each in Brazil, Germany, France, Romania, the Netherlands, and Taiwan. Most studies used data from cohorts or clinical trials.

The risk of bias of the included studies was low (Tables 1-5). The number of patients varied between different studies and groups (Table 6).

**Table 1:** Risk of bias assessment (1/5)

Studies	Kaddurah-Daouk et al., 2011 [10]	Kaddurah-Daouk et al., 2012 [11]	Zheng et al., 2013 [12]	Kaddurah-Daouk et al., 2013 [13]	Zhu et al., 2013 [14]	Zheng et al., 2013 [15]	Ding et al., 2014 [16]	Liu et al., 2015 [17]	Moaddel et al., 2015 [18]	Setoyama et al., 2016 [19]	Zheng et al., 2016 [20]
STROBE items											
Title and abstract												X	X	X	X	X	X	X	X		X	X
Background/rationale	X	X	X	X	X	X	X	X	X	X	X
Objectives	X	X	X	X	X	X	X	X	X	X	X
Study design	X	X	X	X	X	X	X	X	X	X	X
Setting			X		X	X	X	X	X	X	
Participants	X	X	X	X	X	X		X	X		X
Variables	X	X	X			X	X	X		X	X
Data source/measurement	X	X	X	X	X	X	X	X	X	X	X
Bias							X				
Study size	X	X	X	X	X	X		X	X	X	
Quantitative variables	X	X	X	X	X	X	X	X	X	X	X
Statistical methods	X	X	X	X	X	X	X	X	X	X	X
Participants						X	X			X	
Descriptive data	X	X	X	X	X	X	X	X	X		X
Outcome data	X	X	X	X	X	X	X	X	X	X	X
Main results	X	X	X	X	X	X	X	X	X	X	X
Other analysis		X				X	X	X	X	X	X
Key results	X	X	X	X	X	X	X	X	X	X	X
Limitations	X			X	X	X	X			X	X
Interpretation	X	X	X	X	X	X	X	X	X	X	X
Generalizability	X	X	X	X	X	X		X		X	X
Funding	X	X	X		X	X	X	X	X	X	X
Total	18	18	18	16	18	21	19	19	16	19	18

**Table 2:** Risk of bias assessment (2/5)

Studies	Rotroff et al., 2016 [21]	Liu et al., 2016 [22]	Ali-Sisto et al., 2016 [23]	Zheng et al., 2016 [24]	Chen et al., 2017 [25]	Kageyama et al., 2017 [26]	Zheng et al., 2017 [27]	Chen et al., 2018 [28]	Moaddel et al., 2018 [29]	Pan et al., 2018 [30]	Kawamura et al., 2018 [31]
STROBE items											
Title and abstract												X	X		X	X	X	X	X	X	X	X
Background/rationale	X	X	X	X	X	X	X	X	X	X	X
Objectives	X	X	X	X	X	X	X	X	X	X	X
Study design	X	X	X	X	X	X	X		X	X	X
Setting		X	X		X	X	X	X		X	X
Participants	X		X	X		X	X	X	X	X	X
Variables	X	X	X	X	X	X	X	X	X	X	X
Data source/measurement	X	X	X	X	X	X	X	X	X	X	X
Bias		X	X								
Study size	X	X	X			X	X	X			X
Quantitative variables	X	X	X	X	X	X	X	X	X	X	X
Statistical methods	X	X	X	X	X	X	X	X	X	X	X
Participants		X	X	X				X			X
Descriptive data	X	X	X	X	X	X	X	X		X	X
Outcome data	X	X	X	X	X	X	X	X	X	X	X
Main results	X	X	X	X	X	X	X	X	X	X	X
Other analysis	X	X	X	X	X	X		X	X	X	X
Key results	X	X	X	X	X	X	X	X	X	X	X
Limitations	X		X	X	X	X	X	X	X	X	X
Interpretation	X	X	X	X	X	X	X	X	X	X	X
Generalizability				X	X	X	X	X		X	X
Funding	X	X	X	X	X	X	X	X	X	X	X
Total	18	19	20	19	19	20	19	20	16	19	21

 

**Table 3:** Risk of bias assessment (3/5)

Studies	Liu et al., 2018 [32]	Ali-Sisto et al., 2018 [33]	Liu et al., 2018 [34]	Gui et al., 2018 [35]	Czysz et al., 2019 [36]	Bhattacharyya et al., 2019 [37]	Bhattacharyya et al., 2019 [38]	Chen et al., 2019 [39]	Ahmed et al., 2020 [40]	Brunoni et al., 2020 [41]	Han et al., 2020 [42]
STROBE items											
Title and abstract													X	X	X	X	X	X	X	X	X	
Background/rationale	X	X	X	X	X	X	X	X	X	X	X
Objectives	X	X	X	X	X	X	X	X	X	X	X
Study design	X	X	X	X	X	X	X	X	X	X	X
Setting	X	X	X	X	X	X	X			X	X
Participants	X	X	X	X	X		X			X	X
Variables	X	X	X		X	X	X	X	X	X	X
Data source/measurement	X	X	X	X	X	X	X	X	X	X	X
Bias				X	X					X	X
Study size	X	X	X	X	X	X	X			X	X
Quantitative variables	X	X	X	X	X	X	X	X	X	X	X
Statistical methods	X	X	X	X	X	X	X	X	X	X	X
Participants		X	X		X					X	X
Descriptive data	X	X	X	X	X		X	X	X	X	X
Outcome data	X	X	X	X	X	X	X	X	X	X	X
Main results	X	X	X	X	X	X	X	X	X	X	X
Other analysis	X	X	X	X	X	X	X	X	X	X	X
Key results	X	X	X	X	X	X	X	X	X	X	X
Limitations		X	X	X	X	X	X	X	X	X	X
Interpretation	X	X	X	X	X	X	X	X	X	X	X
Generalizability			X	X	X	X		X	X	X	X
Funding	X	X	X	X	X	X	X	X	X	X	X
Total	17	20	21	21	22	18	19	17	17	22	21

 

**Table 4:** Risk of bias assessment (4/5)

Studies	Erabi et al., 2020 [43]	Zhao et al., 2020 [44]	Shen et al., 2020 [45]	Du et al., 2021 [46]	Gamradt et al., 2021 [47]	Homorogan et al., 2021 [48]	Tateishi et al., 2021 [49]	Caspani et al., 2021 [50]	Hung et al., 2021 [51]	Bai et al., 2021 [52]	Kageyama et al., 2021 [53]
STROBE items											
Title and abstract												X	X	X	X		X	X	X	X		X
Background/rationale	X	X	X	X	X	X	X	X	X	X	X
Objectives	X	X	X	X	X	X	X	X	X	X	X
Study design	X	X	X	X	X	X	X	X	X	X	X
Setting		X					X	X	X		
Participants	X	X	X	X	X	X	X	X	X	X	X
Variables	X	X	X	X	X	X	X	X	X		X
Data source/measurement	X	X	X	X	X	X	X	X	X	X	X
Bias				X				X		X	
Study size	X	X						X			
Quantitative variables	X	X	X	X	X	X	X	X	X	X	X
Statistical methods	X	X	X	X	X	X	X	X	X	X	X
Participants	X							X			X
Descriptive data	X	X	X	X	X	X	X	X	X		X
Outcome data	X	X	X	X	X	X	X	X	X	X	X
Main results	X	X	X	X	X	X	X	X	X	X	X
Other analysis	X		X	X	X	X	X	X	X	X	X
Key results	X	X	X	X	X	X	X	X	X	X	X
Limitations	X			X	X	X	X	X	X	X	X
Interpretation	X	X	X	X	X	X	X	X	X	X	X
Generalizability	X				X			X		X	
Funding	X	X	X	X	X	X	X	X	X	X	X
Total	20	17	16	18	17	17	18	22	18	16	18

 

**Table 5:** Risk of bias assessment (5/5)

Studies	Mocking et al., 2021 [54]	Brydges et al., 2021 [55]	Ciocan et al., 2021 [56]	Kurokawa et al., 2021 [57]	Hu et al., 2021 [58]	Joyce et al., 2021 [59]
STROBE items						
Title and abstract								X	X	X	X	X
Background/rationale	X	X	X	X	X	X
Objectives	X	X	X	X	X	X
Study design	X	X	X	X	X	X
Setting		X	X	X	X	
Participants	X	X	X	X	X	X
Variables	X	X	X	X	X	X
Data source/measurement	X	X	X	X	X	X
Bias		X		X		X
Study size			X		X	X
Quantitative variables	X	X	X	X	X	X
Statistical methods		X		X	X	X
Participants	X	X	X	X	X	
Descriptive data	X	X	X	X	X	X
Outcome data	X	X	X	X	X	X
Main results	X	X	X	X	X	X
Other analysis	X	X	X	X	X	X
Key results	X	X	X	X	X	X
Limitations	X	X	X	X	X	X
Interpretation	X	X	X	X	X	X
Generalizability	X	X				X
Funding	X	X	X	X	X	X
Total	17	21	19	20	20	20

 

**Table 6:** Summary of 50 studies evaluating the metabolomics of MDD Abbreviations: ↑ - high levels; ↓ - low levels; BCAA - branched chain amino acids; BD - bipolar depression; BDI - Beck Depression Inventory; CBT - cognitive behavioral therapy; CCMD-3 - Chinese Classification of Mental Disorders, Third Edition; CE - capillary electrophoresis; CES-D - Center for Epidemiologic Studies Depression Scale; CIS-R - Clinical Interview Schedule-Revised, CSF - cerebrospinal fluid; DFI - direct flow injection; DSM-IV - Diagnostic and Statistical Manual of Mental Disorders, Fourth Edition;  DSM-5 - Diagnostic and Statistical Manual of Mental Disorders, Fifth Edition; EPDS - Edinburgh Postnatal Depression Scale; FIA - flow injection analysis; GC - gas chromatography; GlycA - glycoprotein acetylation; HAM-D - Hamilton Depression Rating Scale; HBV - hepatitis B virus; HDRS-17 - the original version of the HAM-D, with only 17 items; HILIC - hydrophilic interaction liquid chromatography; HIV - human immunodeficiency virus; LC - liquid chromatography; LCECA - liquid chromatography with electrochemical coulometric array; LDL - low density lipoprotein; MADRS - Montgomery–Åsberg Depression Rating Scale; MDD - major depressive disorder; MINI - Mini International Neuropsychiatric Interview; MRM - multiple reaction monitoring; MS - mass spectrometry; NMR - nuclear magnetic resonance spectroscopy; PHQ-9 - Patient Health Questionnaire-9; QQQ - triple quadrupole; rTMS - repetitive transcranial magnetic stimulation; SCID-1 - Structured Clinical Interview for DSM-IV Axis I Disorders; SDS - Self-rating Depression Scale; SSRI - selective serotonin reuptake inhibitor; TRD - treatment-resistant depression; TOFMS - Time-of-flight mass spectrometry;  UPLC - ultra performance liquid chromatography; UHPLC-Q-TOF-(ESI+)-MS - ultra-high-performance liquid chromatography coupled with electrospray ionization quadruple time-of-flight mass spectrometry; VLDL - very low density lipoprotein; WEMWBE - Warwick-Edinburgh Mental Well Being Scale.

First author/year	Population/treatment	Method for MDD diagnosis/symptoms assessment	Bodily fluid	Analysis technique	Relevant differences in metabolites
Kaddurah-Daouk et al., 2011 [10]	43 MDD patients treated with sertraline	DSM-IV and HAM-D	Serum	LCECA	Responders in both groups:
					↑ Dihydroxyphenylacetic acid, 4-hydroxyphenyllactic acid, serotonin and gamma tocopherol
	46 MDD patients treated with placebo				
Kaddurah-Daouk et al., 2012 [11]	14 MDD patients	DSM-IV and HAM-D	CSF	Electrochemistry-based metabolomics platform	Recovered from MDD:
					↑ methionine
	14 patients recovered from MDD				↓ 5-Hydroxyindoleacetic acid, 5-Hydroxyindoleacetic acid /tryptophan, 5-hydroxyindoleacetic acid /kynurenine, homovanillic acid, homovanillic acid/ tyrosine, glutathione/ methionine
	18 healthy controls				
Zheng et al., 2013 [12]	82 MDD patients at the first episode	DSM-IV and HAM-D	Urine	NMR	MDD:
	82 healthy controls				↑ alanine, citrate, formate, glycine, isobutyrate, methylmalonate, nicotinate, succinate, taurine, and -ketoglutarate
	Validation:				
	44 MDD patients				↓ 3,4-dihydroxymandelate, choline, creatinine, dimethylamine, dimethylglycine, glyceroylphosphocholine, hippurate, malonate, m-hydroxyphenylacetate, N-methylnicotinamide, phenylacetyglycine, p-hydroxyphenylacetate, and trimethylamine-N-oxide
	52 healthy controls				
Kaddurah-Daouk et al., 2013 [13]	89 first episode MDD patients randomized	DSM-IV and HAM-D	Serum	MS	Sertraline group:
	43 patients -sertraline 50-150 mg				↑ Aconitic acid , cysteine
	46 patients - placebo				↓ Linoleic acid, palmitic acid, arachidonic acid, oleic acid, palmitoleic acid and heptadecanoic acid , glycerol, ornithine, citrulline, xanthine, 5-methoxytryptamine, 3-hydroxybutanoic acid
Zhu et al., 2013 [14]	75 MDD patients randomized	HDRS-17	Serum	LC/GC-MS	Sertraline group:
	35 patients- sertraline 50-150 mg				↑ Quinurenine, 3-hydroxyquinurenine
	40 patients - placebo				↑ 5 methoxytriptofol, melatonin in responders
					↓ 5 –hydroxytryptophan, 5 hidoxindolacetic, 5-methoxytryptamine
Zheng et al., 2013 [15]	21 MDD patients with previous suicide attempt	DSM-IV and HAM-D	Plasma	NMR	Suicide attempters X healthy controls:
	35 MDD patients who never attempted suicide				↑ low-density lipoprotein (LDL), very low-density lipoprotein (VLDL), glucose, acetone, and taurine
	35 healthy controls				↓ cholesterol, unsaturated lipid, pyruvate, lactate, acetate, alanine, valine, glycine, and glutamine
					MDD suicide attempters X MDD nonattempters:
					↑ myo-inositol, glucose, pyruvate, alanine, glycine, and taurine
					↓ low-density lipoprotein (LDL), very low-density lipoprotein (VLDL), cholesterol, unsaturated lipid, and lactate
Ding et al., 2014 [16]	23 MDD patients with early stress	DSM-IV	Plasma	GC-MS	MDD:
	23 MDD patients without early stress				↑ galactose, sorbitol glycine, alanine, proline, serine and butanedioic acid
	25 healthy controls				↓ linoleic acid, oleic acid, heptadecylic acid, myoinositol, mannose, leucine, erythronic acid and cholesterol
Liu et al., 2015 [17]	60 untreated patients on first episode of MDD	DSM-IV and HAM-D	Plasma	LC-MS	MDD:
	59 healthy controls				↑ Triglycerides, phosphatidylcholines, phosphatidylethanolamine, phosphatidylcholines, lyso-phosphatidylcholines, lysophosphatidylethanolamines, taurochenodeoxycholate glycodeoxycholate, and glycoursodeoxycholic acid
					↓ Acyl carnitines, phospholipid, tryptophan, methionine, free fatty acids, lithocholic acid and deoxycholic acid
Moaddel et al., 2015 [18]	21 patients with treatment-resistant MDD:	DSM-IV and MADRS	Plasma	LC-MS	Treatment-resistant MDD:
	8 responders to ketamine and				↑ D-serine, L-serine
	13 non-responders				KET- non-responders > KET- responders > Healthy controls
					Setoyama et al., 2016 [19]	77 MDD patients	HAM-D and PHQ-9	Plasma	LC-MS	↑ 3-hydroxybutyrate, betaine, citrate, creatinine and gamma-aminobutyrate - directly related to the severity of MDD
Zheng et al., 2016 [20]	43 women in the first episode of MDD (19 medicated)	DSM-IV and HAM-D	Urine	NMR; LC-MS and GC-MS							Women with MDD:
	48 healthy female controls				↑m-hydroxyphenylacetate, malonate, isobutyrate, azelaic acid
					↓ glycolate, hypoxanthine
					Men with MDD:
	50 men in the first episode of MDD (12 medicated)				↑citrate and succinate
	75 healthy male controls				↓ tyrosine, n-acetylgluosamine, n-methylnicotinamide
Rotroff et al., 2016 [21]	75 MDD patients randomized to:	DSM-IV and MINI	Plasma	GC	Metabolites associated with increased response to treatment with ketamine: Ornithine, citrulline, tryptophan/kynurenine
	Ketamine n: 33 X				
	Placebo n: 12				
	Esketamine n: 20 X				
	Placebo n: 10				
Liu et al., 2016 [22]	90 MDD patients	HAM-D and MINI	Plasma	GC-MS and LC-MS/MS	MDD:
	97 healthy controls				↑cortisol, androstenedione, corticosterone, dopamine, L-metanephrine, L-normetanephrine, triglycerides and fatty acids
					↓ histamine, arachidonic acid, serotonin
						Ali-Sisto et al., 2016 [23]	99 patients with MDD at the beginning of the study	DSM-IV	Plasma	UPLC-MS	MDD:
↑ xanthine and adenosine										
↓ inosine and guanosine										
	73 followed up until the end									
	253 healthy controls									
Zheng et al., 2016 [24]	Cohort 1	HDRS-17	Plasma	GC-MS	MDD:
	50 individuals with MDD				↑ octanoic acid, hydroxylamine, benzoic acid, γ- aminobutyric acid, homoserine
	50 healthy controls				↓ malonic acid, isoleucine, lanosterol, valine, sorbitol, creatinine, ribulose 5-phosphate, ethanolamine, malic acid, fumaric acid, γ-tocopherol and dopamine
	Cohort 2				
	58 patients with MDD (6 non-medicated and 52 medicated)				
	56 healthy controls				
Chen et al., 2017 [25]	59 patients with moderate MDD compared with 82 healthy controls	DSM-IV and HAM-D	Urine	NMR e GC-MS	Moderate MDD:
					↑ fructose, nicotinate, citrate, isobutyrate, ribose, vanillic acid, sorbitol and azelaic acid
					↓ trimethylamine n-oxide, n- methylnicotinamide, acetone, choline, malonate and glyceroylphophocholine
						Severe MDD:
	34 patients with severe MDD compared with 41 healthy controls				↑ nicotinate, p-hydroxyphenylacetate, sucrose, alanine, taurine, choline, citrate, hydroxylamine, myristic acid, formate, isobutyrate, palmitic acid, lactate and glycine
					↓ α-ketoglutarate, trimethylamine n-oxide, indoxyl sulphate, m-hydroxyphenylacetate, malonate, 3-hy- droxyphenylacetic acid, n-methylnicotinamide and oxalacetate
	Kageyama et al., 2017 [26]				Cohort 1: 9 MDD patients, 19 healthy controls	MINI, DSM-IV and HAM-D	Plasma	GC-TOFMS	MDD: ↑ nervonic acid
Cohort 2: 45 medicated MDD patients, 90 healthy controls									
Zheng et al., 2017 [27]		72 untreated patients on first episode of MDD	HDRS-17	Plasma	NMR					MDD:
	54 healthy controls	↑ Polyunsaturated fatty acids, acetoacetate, VLDL / LDL, adipic acid, glycoproteins, β-glucose and α-glucose, adipic acid			
		↓ Pyruvate and formate			
Chen et al., 2018 [28]	32 healthy controls and 32 patients with MDD and anxiety;	DSM-IV and HAM-D	Urine	GC/MS e NMR.	MDD:
	16 healthy controls and 16 patients with MDD and anxiety				↑ Azelaic acid, aminomalonic acid, (S)-3- hydroxyisobutyric acid, fructose, sorbitol, L-lactic acid, glycine, L-alanine, citric acid, adipic acid, L-threonine, (S)- 3,4-dihydroxybutyric acid, α-aminobutyric acid, and ribose
					↓ Acetone, methylmalonic acid, pseudouridine, indican, hippuric acid and N-methylnicotinamide
						Moaddel et al., 2018 [29]	29 MDD patients randomized to 0.5mg/kg of ketamine or placebo in a crossover design;	DSM-IV and MADRS	Plasma	LC/MS	Baseline:
MDD:										
↑ Kynurenine/tryptophan, serine, tryptophan										
25 healthy controls	↓ Threonine, citrulline									
	Post-infusion:									
	MDD:									
	Kynurenine/tryptophan ↑ at 230 min ↓ day 3 after placebo									
	Kynurenine/tryptophan ↓ at 230 min, ↑day 1, ↓ day 3 after ketamine									
	Healthy controls:									
	Trans-4-hydroxy proline ↑after placebo									
	α-amino adipidic acid ↓ after ketamine									
Pan et al., 2018 [30]	1st cohort: 50 MDD patients and 50 healthy controls;	DSM-IV and HAM-D	Plasma	GC-MS e LC-MS/MS.	MDD:
	2nd cohort: 40 MDD patients, 30 BD patients and 40 healthy controls				↑ γ-aminobutyric acid, tyramine and dopamine
					↓ succinic acid, α-ketoglutaric acid, glutamine, L-tyrosine, tryptophan, and kynurenine
Kawamura et al., 2018 [31]	34 MDD patients	DSM-IV and SCID-I	Plasma	CE-MS	MDD:
					↓ Phosphoethanolamine, taurine, aspartic acid, tyrosine, methionine, asparagine, glycerophosphocholine, hypotaurine, ATP, ADP, histidine, lysine, phenylalanine 1, 2-aminoadipic acid
						31 healthy controls
	Liu et al., 2018 [32]					290 MDD patients	HAM-D	Plasma	LCECA	↓ Kynurenine was associated with more severe depressive symptoms
Ali-Sisto et al., 2018 [33]		78 medicated MDD patients	DSM-IV	Plasma	UPLC-MS						MDD:
											↓ Arginine and dimethyl arginine
	253 healthy controls										
Liu et al., 2018 [34]	50 MDD patients	DSM-5 and HAM-D-24	Serum and urine	GC-MS	MDD:
					Serum:
					↑ l-valine, l-lysine, l-leucine
					Urine:
	28 healthy controls				↑ N-acetyld-glucosamine, stearic acid, threonic acid ↑
Gui et al., 2018 [35]	20 MDD patients	DSM-IV and HAM-D	Plasma	LC-MS/ MS	MDD:
					↑ LDL, lysophospholipid, IL-6, TNF
	20 healthy controls				↓ Phospholipids, apolipoprotein E, haptoglobin serotransferrin, apolipoprotein A-5, complement factor H and immunoglobulin gamma, HDL
Czysz et al., 2019 [36]	159 MDD patients randomized to three groups (8-12 weeks)	HDRS-17	Plasma	LC e FIA - MS	↑ hydroxyphingomyelin / sphingomyelin were associated with better response to antidepressant treatment in the three groups
	Escitalopram + Placebo X				
	Escitalopram + Bupropion X				
	Venlafaxine + Mirtazapine				
						Bhattacharyya et al., 2019 [37]	290 MDD patients treated with citalopram, escitalopram or other SSRI	HAM-D	Plasma	LCECA	Post-treatment:
↑ 5-Hydroxyindoleacetic acid / serotonin, Indole-3-acetic, Vanillylmandelic, 4-Hydroxyphenylacetic , 4-Hydroxyphenylacetic acid/ Tyrosine, 4-hydroxybenzoic acid, Paraxanthine / Xanthine and Uric acid / Xanthine										
↓ Serotonin, Methoxy-hydroxyphenyl glycol , Methoxy-hydroxyphenyl glycol /Tyrosine, Hypoxanthine, Xanthine, Xanthine / Xanthosine										
Bhattacharyya et al., 2019 [38]	26 MDD patients randomized for CBT (subgroup analysis)	DSM-IV and HAM-D	Serum	UPLC / MS / MS	Baseline:
					Remitters: ↑ phosphatidylcholines
					Non-responders compared to remitters:
					↑ Acylcarnitines, α-aminoadipic acid, phenylalanine, tyrosine and tryptophan
					Over the course of treatment:
					Remitters: ↑ phosphatidylcholines
					Non-responders:
					↓ phosphatidylcholines
Chen et al., 2019 [39]	Young	DSM-IV and HAM-D	Urine	NMR and GC-MS	Young and middle aged MDD:
	(18-29 years):				↑ Citric acid and oxoglutaric
	44 MDD patients				↓ Hypoxanthine, indoxyl sulfate, pseudouridine, quinolinic acid, l tyrosine, 1 metthylinosine, uracil, ethanoloamine
	56 healthy controls				
	Middle age				
	(30-59 years):				
	74 MDD patients				
	61 healthy controls				
Ahmed et al., 2020 [40]	240 MDD patients treated with citalopram or escitalopram for eight weeks	HDRS-17	Plasma	UPLC/MS	Over the course of treatment:
					↑short-chain acylacrnitines
					↓ medium and long- chain acylacrnitines
Brunoni, et al., 2020 [41]	4364 patients	CIS-R	Plasma	NMR profiler platform	↑ GlycA levels were associated with persistent depression
	No depression (n = 4024)				↑ baseline GlycA levels were associated with worsening of depressive symptoms
	Incident depression (n = 159, 3.64%)				
	Remitted depression (n = 133, 3.05%)				
	Persistent depression (n = 48, 1.1%)				
Han et al., 2020 [42]	165 MDD patients	PHQ-9 and WEMWBS	Plasma	LC-MS	MDD:
	130 subclinical low mood controls				↑ α- 1-acid glycoprotein 1, leucine-rich α-2-glycoprotein, apolipoprotein E, complement factor H
					↓ retinal dehydrogenase 1
Erabi et al., 2020 [43]	88 MDD patients	DSM-IV, MINI, and HAM-D	Plasma	LC-MS	MDD at baseline:
					↑ 5-oxoproline, 3-hydroxybutyrate, nicotinamide, glutamate and putrescine
	(62 completed approximately six-week treatment with escitalopram)				↓sarcosine, serine, alanine, xanthurenate, xanthosine, tyrosine, phenylalanine, 3-methylhistidine, asparagine, kynurenic acid, 2-aminois- ovaleric acid, threonine, tryptophan, pyruvate and 3-hydroxykynurenine
	88 healthy controls				↓ kynurenic acid and kynurenine were associated with a better therapeutic response to escitalopram
Zhao et al., 2020 [44]	12 MDD female students	BDI-II and SDS	Urine	LC-MS	MDD:
	12 healthy female students				↑ malonic acid, fumaric acid, 2-methylfuma- rate, L-malic acid, and palmitic acid
					↓ 4-acetamidobutyric acid, α-ketoglutaric acid, tartaric acid, gluconic acid, sphingosine, and 21-hydroxypregnenolone
Shen et al., 2020 [45]	120 MDD patients analyzed pre and post treatment with fluoxetine for eight weeks	DSM-IV	Serum	UPLC-Q-TOF/MS	Untreated MDD:
					↑ D-Aspartic acid, CoA, D-Glucose, ADP, Citric acid, Phenylpyruvic acid, Tyrosine, 5-Hydroxyindoleacetaldehyde, Oxoglutaric acid and N-Acetylneuraminic acid
					↓ Lyso PC(O-18:0), Androsterone, Lyso PC(20:1(11Z)), Lyso PC(P-18:1(9Z)), Acetyl-CoA and Thromboxane B2
					Post-fluoxetine MDD:
					↑ Lyso PC(O-18:0), Androsterone, Lyso PC(20:1(11Z)), Lyso PC(P-18:1(9Z)), Acetyl-CoA and Thromboxane B2
					↓ D-Aspartic acid, CoA, D-Glucose, ADP, Citric acid, Phenylpyruvic acid, Tyrosine, 5-Hydroxyindoleacetaldehyde, Oxoglutaric acid and N-Acetylneuraminic acid
Du et al., 2021 [46]	53 MDD patients	DSM-IV	Plasma	LC-MS/MS	MDD:
	83 healthy controls				↓ gamma-glutamyl leucine, leucine-enkephalin, and valeric acid
Gamradt et al., 2021 [47]	28 MDD patients	DSM-5 and MINI	Plasma	LC-MS and GC-MS	MDD:
	28 healthy controls				↑ LDL/HDL ratio
Homorogan et al., 2021 [48]	11 MDD patients treated with escitalopram for 12 weeks	DSM-IV-TR and HAM-D	Plasma	UHPLC-Q-TOF-(ESI+)-MS	MDD at baseline vs. controls:
					↑ phosphatidylserine (16:0/16:1) and phosphatidic acid PA (18:1/18:0)
					MDD after treatment vs. at baseline:
	11 healthy controls				↓ phosphatidylserine (18:3/20:4)
Tateishi et al., 2021 [49]	13 patients with TRD subjected to high frequency rTMS	DSM-5,HAM-D and BDI	Plasma	LC-MS	All patients:
					↑ tryptophan, ↓ serotonin
					rTMS nonresponders: ↑ 5-hydorxytryptophan
					Increase in kynurenine correlated with increased BDI scores.
Caspani et al., 2021 [50]	211 MDD patients treated with escitalopram, augmented with aripiprazole if non-responders (97)	MINI and MADRS	Plasma	NMR spectroscopy	MDD:
					↑ LDL, triglycerides, cholesterol, free cholesterol, phospholipids, apolipoprotein B
					Apolipoprotein A1, HDL Apolipoprotein A1 and HDL 3 free cholesterol presented a negative correlation with a reduction in MADRS score in phase I
						112 healthy controls
	Hung et al., 2021 [51]					229 MDD patients	DSM-IV and HAM-D	Plasma	NMR	MDD in full remission:
67 healthy controls		↑ histidine							
After 10 years:		↓ succinic acid, proline, acetic acid, creatine, glutamine, glycine and pyruvic acid							
137 attended follow-up									
47 full remission									
Bai et al., 2021 [52]	60 MDD patients	DSM-IV and HAM-D	Plasma	LC-MS	MDD:
	60 healthy controls				↑ uridine triphosphate, benzoic acid, 1Heptadecanoyl
					↓ Arachidonic acid, Chenodeoxycholic acid, Deoxycholic acid, Docosahexaenoic acid, 1, Taurocholic acid, Taurochenodeoxycholic Acid
					Inflammation-associated metabolites:
					Arachidonic acid, Chenodeoxycholic acid, Docosahexaenoic acid, Taurochenodeoxycholic Acid, Taurocholic acid, Ethylmethylacetic acid, Deoxyglycocholic acid
Kageyama et al., 2021 [53]	30 MDD patients	DSM-IV and HAM-D	CSF	GC-MS	Nervonic acid levels did not differ among the patients with MDD and healthy controls
	30 healthy controls				
Mocking et al., 2021 [54]	Recurrent MDD in drug free remission:	DSM-IV and HAM-D	Plasma	GC-MS	Predictors of recurrence:
	45 females; 23 males				Females:
					↑ lysophosphatidylcholine 16:0, arachidonic acid, DHEA-S
	Recurrence:				↓ methylcysteine, monohexosylceramide, glutamine, histidine, ceramides
	24 Females; 11Males				
					Male:
					↑ allantoin, cytosine, alanine, imidazoleacetic acid
	Never depressed controls:				
	40 females; 19 males				↓ 15 hydroxyeicosatetraenoic acid, beta carotene
	Monitored for 2.5 years				
Brydges et al., 2021 [55]	196 MDD patients,	DSM-IV and HAM-D	Plasma	GC-MS	MDD:
	124 available at week 12:				↑ indole metabolites
	34 CBT;				Medication-treated patients:
	44 duloxetine;				↑ IPA (indole-3-propionic acid) and ILA (indole-3-lactic acid)
	46 escitalopram				↓ IAA (indole-3-acetic acid)/IS (Indoxyl sulfate) ratio and IAA/IPA ratio
					CBT-treated patients:
					↑ IAA/IPA ratio
					Remitters to medication:
					↑ IPA, ↓ ILA/IPA
					Remitters to CBT:
					↓ IPA/IS
Ciocan et al., 2021 [56]	56 MDD patients treated with:	DSM-IV-TR	Blood	LC-MS/MS	MDD at baseline
	venlafaxine (25); citalopram (19); or escitalopram (12)				↑ L-serine
	56 healthy controls				↓ aspartic acid and kynurenine levels
					MDD post-treatment
					↑ L-tyrosine, N-acetylornithine and kynurenine
					↓ L-isoleucine
Kurokawa et al., 2021 [57]	33 MDD patients:	DSM-5	Fecal	CE-TOF-MS	No difference was observed post correction
	11 responders to medication; 16 non-responders; 6 stable remitters				
Hu et al., 2021 [58]	144 MDD patients were randomized to ShenZhiLing (73) or fluoxetine (71) and were treated for 8 weeks	DSM IV	Blood	Western blot	Fluoxetine group:
					↓ ApoB/ApoA
					There was statistical difference in ApoC3 between the two groups at the end of the treatment
Joyce et al., 2021 [59]	298 MDD patients	HAM-D	Blood	MS	
	Citalopram (112) Escitalopram (152) Escitalopram + Placebo (34)				Baseline ratio of hydroxylated to non-hydroxylated sphingomyelins, as well as a larger change in this ratio by therapy, predicted greater reduction in depressive symptoms
	298 MDD patients				
	Venlafaxine + Mirtazapine (42) Escitalopram + Bupropion (35)				

Of the studies evaluated in our review, 21 evaluated therapeutic approaches [10,13,14, 18-21,26,29,33,36-38,40,43,48-50,55,56,58]. One study assessed arms with bupropion, venlafaxine, and mirtazapine [36]. Three studies found differently expressed metabolites after treatment with ketamine or s-ketamine [18,21,29]. One study used cognitive behavioral therapy to compare the metabolomic profile at baseline and during treatment [37], while another evaluated the effects of repetitive transcranial magnetic stimulation [49].

The other studies compared MDD individuals with and without medication or MDD patients with healthy controls, but not all studies specified which drugs were used (Table 2) [11,16,19,20,26-28,33,39,51]. One study that used fecal samples did not find any difference in metabolomic profile after correction of statistical analyses [57]. The remaining studies did not assess the interference of a specific therapy on metabolomic profile. The variety of methodologic and metabolic profiles hinders a precise conclusion of the effects of treatment on metabolites. However, the studies suggest possible biomarkers as predictors for the treatment of depression.

Many metabolites were related to MDD in the analyzed studies, and the methods used for detection were heterogeneous, with emphasis on liquid chromatography and gas chromatography associated with mass spectrometry and nuclear magnetic resonance spectroscopy. The fluids evaluated were plasma, serum, urine and cerebrospinal fluid, and for purposes of description, the metabolites were classified according to the fluids in which they were evaluated and their molecular characteristics.

Plasma/serum

Lipids

Sphingomyelin, a sphingolipid that has a structural and cellular signaling function and is abundant in nerve tissues (particularly in myelin), is a fundamental component of cell membranes, and a high hydroxysphingomyelin/sphingomyelin ratio has proven to be a predictor of a good response to antidepressant treatment [36,59]. Patients with depression treated with intravenous ketamine had a sphingomyelin serum level increase at the time-point of 230 minutes after infusion. On the third day after ketamine infusion, the sphingomyelin levels appeared to decrease [29].

Arachidonic acid is an essential fatty acid and a major component of the cell membrane. In MDD patients, arachidonic acid plasma levels appear to be lower than in healthy subjects [22,52]. Treatment with sertraline is implicated in the augmentation of arachidonic acid in MDD patients, which is associated with a reduction in depressive symptoms [13]. High levels of arachidonic acid were predictors of recurrence [54]. Additionally, one study showed lower levels of linoleic, oleic, and heptadecylic acid and cholesterol in MDD patients with early stress [16].

Additionally, valeric acid was also found to be reduced in MDD patients, which could be explained by dysregulation of the brain-gut-microbiota axis or increased N-methyl-d-aspartate (NMDA) receptor activity [46]. On the other hand, nervonic acid, a monounsaturated fatty acid important for myelin synthesis, has been elevated in patients with MDD [26] but cerebrospinal fluid nervous acid levels did not differ between MDD patients and healthy controls [53].

A higher LDL/HDL ratio was found in MDD patients along with a reduction in omega-3 fatty acids levels [47,60] and a positive correlation between the Montgomery-Åsberg Depression Rating Scale (MADRS) score and serum levels of LDL, triglycerides, cholesterol, free cholesterol, phospholipids and apolipoprotein B before treatment [50]. Furthermore, a study that compared eight weeks of treatment with fluoxetine found reduced ApoB (lipoprotein B)/ApoA1 (lipoprotein A1) [58]. Additionally, Zheng et al. compared patients untreated in their first episode of MDD with healthy controls and found higher levels of polyunsaturated fatty acids and VLDL/LDL ratio in the MDD group. Low levels of cholesterol were present in MDD patients [16].

Amines

Phosphoethanolamine is a precursor to cell membrane phospholipids and is related to stages of cell metabolism such as apoptosis, which is why it has been studied in oncology and other areas [61]. A study suggests phosphoethanolamine as a possible biomarker for depression, as lower levels were found in MDD patients compared to healthy controls. However, the small sample size limited the comparison between MDD individuals undergoing pharmacological treatment and drug-naïve MDD subjects [31].

Higher baseline levels of melatonin, a hormone produced in the pineal gland using tryptophan as a substrate and whose primary function is sleep control, correlated with better response to treatment, and in responders their levels increased more [14].

Neurotransmitters

The main inhibitory neurotransmitter in the central nervous system (CNS), gamma-aminobutyric acid (GABA), as well as dopamine, a CNS modulating neurotransmitter, showed elevated levels in depressed patients [20,22,30]. Differently, histamine, one of the main chemical mediators involved in the allergic inflammatory response, seems to be low in MDD [22]. Leucine-enkephalin, an endogenous opioid with a high affinity for the delta-opioid receptor, was also found to be reduced in MDD patients [46].

Amino Acids

Low levels of tryptophan and kynurenine have been observed in MDD [30,32,56]. Indoles (metabolites of tryptophan) are elevated in MDD patients [55]. An inverse relationship was also observed between serum kynurenine levels and the severity of MDD: the lower the levels, the greater the severity [32]. In responders to an antidepressant, the kynurenine/melatonin and 3-hydroxyquinurenine/melatonin ratios decreased and the metabolite 3-hydroxyquinurenine also contributed to distinguish responders and non-responders [10,14]. Furthermore, antidepressant treatment increased kynurenine levels [56], and increased kynurenine levels were also associated with increased Beck Depression Inventory (BDI) scores after repetitive transcranial magnetic stimulation (rTMS) in treatment-resistant depression [49].

During treatment with intravenous ketamine, there was a slight increase in kynurenine (a tryptophan metabolite necessary for the synthesis of vitamin B3) 230 minutes after treatment. There was also a small reduction in these levels on the third day after this intervention [29]. Ketamine non-responders showed higher levels of D-serine and L-serine when compared with ketamine responders and controls [18]. In treatments with ketamine and esketamine, there was a reduction in metabolites of tryptophan and tyrosine [21].

Isoleucine, an essential amino acid that has direct effects on hypothalamic regulation by increasing satiety, has been linked to MDD when at low levels [13,20,37]. Low levels of gamma-glutamyl leucine were also associated with MDD, which may suggest abnormalities in the function of glutathione, an important antioxidant [46]. Arginine, which is an important amino acid for immune function and wound healing, has its lowest levels in MDD. Additionally, arginine levels increased significantly in recovered patients [33]. Treated patients showed higher levels of L-tyrosine, N-acetylornithine, kynurenine and lower levels of L-isoleucine [56].

Hung et al. showed that, in comparison to healthy controls, patients with MDD in complete remission presented significantly lower levels of metabolites related to pyruvate metabolism, via the tricarboxylic acid (TCA) cycle, linked to the metabolism of amino acids including alanine, aspartate, glutamate, arginine and proline and the metabolites of glycine, serine, and threonine. These findings corroborate those of Diang et al., who had previously reported lower levels of leucine and higher levels of alanine, serine, and proline in patients with MDD, pointing to an important involvement of amino acids in the pathophysiology and treatment response of MDD.

Genetic Products

Inosine, a nucleoside that has been studied as a neuroprotective in pathologies such as stroke, Parkinson's disease, and multiple sclerosis, showed low levels in depressed patients. In contrast, the serum levels of xanthine and adenosine were high in MDD [23,33]. Xanthine is a purine base and many stimulants such as caffeine and theobromine are derived from it [62]. Adenosine, on the other hand, is an endogenous purine that performs some functions in the CNS such as inducing sleep and relieving anxiety symptoms.

Others

5-Hydroxyindoleacetic acid, a metabolite of serotonin, tended to increase with the treatment of MDD, and the reduction of serotonin in the body can trigger emotional instability, insomnia, anxiety, and increased appetite [37].

Acylcarnitine, a hormonal activator that has been studied in pathologies such as Alzheimer's disease, showed low levels in subjects with MDD and elevated levels after treatment [17,40]. In contrast, dopamine, normetanephrine and metanephrine (metabolites of catecholamine) have shown high levels in MDD [22].

Glycoprotein acetylation (GlycA) is a novel inflammatory marker based on protein plasma glycosylation. There was a significant association between baseline GlycA levels and depression persistence. The role of high-sensitivity c-reactive protein (hsCRP), a traditional inflammatory marker, was also investigated, as well as the role of these inflammatory markers in the progression of depressive symptoms. GlycA levels predicted depression persistence. The association was robust and significant in fully adjusted models. Moreover, GlycA was superior to hsCRP in predicting depression persistence [41]. Other glycoproteins such as α-1-acid glycoprotein 1 and leucine-rich α-2-glycoprotein were higher in the MDD group [42]. These findings corroborate the involvement of this molecule in MDD.

Taurine, glycine, lysine, l-lysine, valine, l-valine, proline, l-proline, citrulline, citrate, creatinine, and phospholipids, among other metabolites, have also been associated with MDD or the antidepressant response [13,15,19,31,34,35].

Compared with controls, MDD patients with full remission had significantly lower expression of succinic acid, acetic acid, and pyruvic acid [51].

Urine

Amino Acids

Elevated levels of homocysteine, a sulfhydryl amino acid formed from methionine, are related to neurological and cerebrovascular diseases. Young and middle-aged MDD patients had low levels of l-tyrosine compared with healthy controls [39]. In a study with plasma samples, the results were concordant, with treated patients showing higher levels of l-tyrosine [56].

Acids

Malonate, which is an inhibitor of cellular respiration, has lower levels in MDD [12,25]. It also appeared as a differential metabolite between depressed women and men: while females with MDD presented lower levels of malonate in comparison to healthy subjects, the same was not observed for males. Of note, females with MDD who responded or remitted after antidepressant treatment presented levels of urinary malonate concentration similar to those of healthy participants [20]. Using samples of young and middle-aged MDD patients, higher levels of citric acid and oxoglutaric acid and lower levels of quinolinic acid were found in this population compared to healthy individuals [39]. 

Others

After assessing college students, high concentrations of malonic acid, fumaric acid, 2-methylfuma- rate, L-malic acid, and palmitic acid and lower levels of 4-acetamidobutyric acid, α-ketoglutaric acid, tartaric acid, gluconic acid, sphingosine, and 21-hydroxypregnenolone were capable to differentiate depressed students from healthy students [44]. In a group of patients with MDD and anxiety higher levels of acid azelaic, aminomalonic, (S)-3-hydroxyisobutyric, l-lactic, adipic, (S)-3,4-hydroxyisobutyric and α-aminobutyric acid and low levels of methylmalonic and hippuric acid were capable of differentiated of healthy controls [28].

Cerebrospinal fluid

Amino Acids

MDD patients in remission presented differences in tryptophan and tyrosine metabolism compared to MDD patients without remission and controls. The group in remission also had higher methionine levels and higher methionine/glutathione ratios than the other MDD and control groups, suggesting the involvement of methylation pathways and oxidative stress [11].

Discussion

Metabolomics research is an area of learning that explores metabolic pathways associated with various health problems, helps in understanding the pathophysiology of diseases, including mental disorders, and enables the discovery of biomarkers. This systematic review aimed to identify metabolites that are differentially expressed in MDD. After analyzing the selected studies, we found many metabolites related to MDD diagnosis and/or treatment, acting with cell signalers, cell membrane components, neurotransmitters, inflammatory and immunological mediators, hormonal activators and precursors and sleep controllers. However, there was a wide variation in the analyzed fluids and assessment methods, possibly due to the heterogeneity of metabolites found.

As was expected, compounds from the tryptophan, tyrosine, and purine pathways were differently expressed in MDD patients in many of the reviewed studies, as metabolic factors in the kynurenine pathway are considered possible mechanisms involved in the pathophysiology of MDD. The kynurenine pathway begins with the conversion of tryptophan to kynurenine. For the first branch, kynurenine is transformed into 3-hydroxyanthranilic acid and quinolinic acid, which are N-methyl-d-aspartate (NMDA) receptor agonists that exert neurotoxic effects. For the second branch, kynurenine is transformed into kynurenic acid by kynurenine aminotransferases. Kynurenic acid is an NMDA receptor antagonist, which exerts a neuroprotective effect [43]. Tryptophan is an essential amino acid necessary for the production of serotonin and melatonin [63]. The two main metabolism pathways for tryptophan are 5-hydroxytryptophan and kynurenine.

Lower baseline plasma kynurenine is significantly associated with the severity of depressive symptoms and suicidal ideation. Kynurenine biosynthesis in the brain occurs primarily in astrocytes while tryptophan catabolism occurs mainly in glial cells. Kynurenine can cross the blood-brain barrier. Peripheral kynurenine, primarily generated in the liver, is the source of ∼60% of CNS kynurenine. However, the relationship of plasma kynurenine concentration to MDD symptom severity remains unclear and further investigations are necessary. A toxic kynurenine metabolite acts as an NMDA receptor agonist, which has been linked to depressive symptoms and other psychiatric manifestations [32].

Rotroff et al. also studied this metabolic pathway through a clinical trial with ketamine or esketamine in the treatment of MDD. Esketamine was the most potent enantiomer as an NMDA receptor antagonist. Metabolic changes have been demonstrated in relation to glutamate and tryptophan metabolism. Glutamic acid levels are increased 240 minutes after ketamine exposure. Ketamine is known to block the glutamatergic NMDA receptor; thus, the possible effect of increased glutamate levels could shift glutamatergic signaling from NMDA receptor to AMPA receptor to enhance the 5HT1B receptor activity that is hypothesized to be required for antidepressant effects. Treatment with either ketamine or esketamine resulted in decreased tryptophan metabolites. It is clear that the glutamatergic system appears to contribute to the risk and severity of MDD, requiring further investigation in this regard through original studies.

Another metabolite that deserves to be highlighted in our review is acylcarnitine. Ahmed et al. identified that more severe forms of depression are associated with reductions in short-chain acylcarnitines after SSRI treatment. This finding, and the relationship of acylcarnitine levels with mitochondrial fatty acid β-oxidation and branched-chain amino acid catabolism, suggests that the pathobiology of MDD may manifest, in part, through metabolomic dysfunction. Further, these findings may reflect changes in mitochondrial function or ATP production in patients with MDD. Moaddel et al. found a decrease in acylcarnitine concentrations after ketamine treatment compared to placebo.

Previously published reviews brought together studies that evaluated metabolites in order to identify reliable biomarkers for MDD. In agreement with the present systematic review, Macdonald et al. found a diversity of analyzed fluids and methodologies used. Urine, cerebrospinal fluid, plasma, and serum were also identified as analyzed fluids. Analysis techniques such as the use of gas and liquid chromatography combined with mass spectrometry, capillary electrophoresis time of flight mass spectrometry (CE-TOF-MS), NMR and liquid chromatography with electrochemical coulometric array detection (LCECA) were observed [64]. These techniques are widely used because they allow the simultaneous detection of numerous metabolites [65].

Specific biomarkers were found to be related to MDD by Macdonald et al. (2019), with glycine, alanine, citrate and formate increasing and phenylalanine, valine, aminoethanol, and hypurate is shown to be negatively regulated. Other metabolites were found to be only potentially consistent. Some metabolic pathways were found to be involved in the pathophysiology of MDD such as coenzyme Q biosynthesis, glycine-serine-threonine metabolism, tyrosine metabolism, pyrimidine metabolism, and steroid biosynthesis [44,45,56,66].

Our findings might be helpful to researchers in the field and to future research question formulations, by elucidating which metabolites seem to be associated with MDD pathophysiology, laboratory diagnosis and therapeutic approaches and which, therefore, should be investigated further. It is also important to discover specific molecular biomarkers for mental disorders, as an objective and complementary method to currently existing diagnoses, which use concepts that mostly have a subjective character. The metabolic profile can be used as a response predictor, thus assisting in making more targeted decisions [65].

The experiments considered in the review identified increased or reduced levels of metabolites, but not necessarily increased or reduced activity of the associated pathways. The information acquired through metabolic analyses does not specify whether the changes identified in the metabolites are a cause or a consequence of the pathology. It is not known, therefore, whether the affected pathways in different psychiatric conditions represent causal mechanisms of the diseases. Gadad et al. concluded that no biomarker has been translated into clinical practice for the diagnosis of depression or treatment definition. However, some recent studies have suggested the possibility of diagnostic metabolic panels for depressive disorders, such as phosphatidylserine (16:0/16:1) for MDD, with an AUC value of 0.876 [48].

Some limitations are the heterogeneity of studies on metabolites, methods for detection, analyzed body fluids, and treatments used. Not all studies had detailed assessment tools such as Hazard Ratio. Thus, it was not possible to carry out the synthesis with meta-analysis. To minimize the heterogeneity, we chose to include in this review only patients with unipolar depression, without associated health conditions, such as pregnancy and puerperium, hepatitis, and acquired immunodeficiency syndrome. However, prevalent health conditions such as diabetes mellitus, systemic arterial hypertension, obesity, and dyslipidemia were not detailed in the studies. MDD is not a phenotypically and genetically homogeneous disorder. Rather, MDD might be seen as a highly prevalent syndrome, with a wide polygenicity and present in people of different ages and ethnic backgrounds [67]. Likewise, several gene-environment interactions have been suggested as risk factors of MDD. Because of such heterogeneity, metabolite-mapping related to MDD is a challenge.

As seen in most of the studies cited in this review, MDD metabolite investigation protocols do not take into count that heterogeneity; therefore, their results might be influenced by samples with significantly different phenotypes, severity, ages or treatment approaches. The data obtained were not sufficient to distinguish changes directly related to disease or as a consequence of life habits, phenotypic characteristics and effects related to treatment. Therefore, it would be advantageous for metabolomic studies to investigate more homogeneous MDD subtypes.

## Conclusions

The results presented in our review show that several metabolites are altered in MDD and change with treatment, and the most consistent evidence available relates to kynurenine and acylcarnitine. However, there is still not enough evidence to propose changes in the diagnosis or therapeutic management of MDD. In view of the limitations presented, we suggest the investigation of metabolites in MDD in more homogeneous conditions, taking into consideration both the MDD phenotype and the patient’s characteristics. Considering the benefits that biomarkers can bring to the pathophysiological understanding, diagnosis and treatment of MDD, further metabolomics research is a necessity.

## References

[1] Wang PS, Simon G, Kessler RC (2003). The economic burden of depression and the cost-effectiveness of treatment. Int J Methods Psychiatr Res.

[2] Bot M, Milaneschi Y, Al-Shehri T (2020). Metabolomics profile in depression: a pooled analysis of 230 metabolic markers in 5283 cases with depression and 10,145 controls. Biol Psychiatry.

[3] Humer E, Probst T, Pieh C (2020). Metabolomics in psychiatric disorders: what we learn from animal models. Metabolites.

[4] Nedic Erjavec G, Konjevod M, Nikolac Perkovic M (2018). Short overview on metabolomic approach and redox changes in psychiatric disorders. Redox Biol.

[5] Nicholson JK, Lindon JC (2008). Systems biology: metabonomics. Nature.

[6] Schwarz E, Bahn S (2008). The utility of biomarker discovery approaches for the detection of disease mechanisms in psychiatric disorders. Br J Pharmacol.

[7] Moher D, Liberati A, Tetzlaff J, Altman DG (2009). Preferred reporting items for systematic reviews and meta-analyses: the PRISMA statement. Ann Intern Med.

[8] (2019). Cochrane Handbook for Systematic Reviews of Interventions.

[9] von Elm E, Altman DG, Egger M, Pocock SJ, Gøtzsche PC, Vandenbroucke JP (2008). The Strengthening the Reporting of Observational Studies in Epidemiology (STROBE) statement: guidelines for reporting observational studies. J Clin Epidemiol.

[10] Kaddurah-Daouk R, Boyle SH, Matson W (2011). Pretreatment metabotype as a predictor of response to sertraline or placebo in depressed outpatients: a proof of concept. Transl Psychiatry.

[11] Kaddurah-Daouk R, Yuan P, Boyle SH (2012). Cerebrospinal fluid metabolome in mood disorders-remission state has a unique metabolic profile. Sci Rep.

[12] Zheng P, Wang Y, Chen L (2013). Identification and validation of urinary metabolite biomarkers for major depressive disorder. Mol Cell Proteomics.

[13] Kaddurah-Daouk R, Bogdanov MB, Wikoff WR (2013). Pharmacometabolomic mapping of early biochemical changes induced by sertraline and placebo. Transl Psychiatry.

[14] Zhu H, Bogdanov MB, Boyle SH (2013). Pharmacometabolomics of response to sertraline and to placebo in major depressive disorder - possible role for methoxyindole pathway. PLoS One.

[15] Zheng P, Gao H-C, Qi Z-G (2013). Peripheral metabolic abnormalities of lipids and amino acids implicated in increased risk of suicidal behavior in major depressive disorder. Metabolomics.

[16] Ding X, Yang S, Li W (2014). The potential biomarker panels for identification of Major Depressive Disorder (MDD) patients with and without early life stress (ELS) by metabonomic analysis. PLoS One.

[17] Liu X, Zheng P, Zhao X (2015). Discovery and validation of plasma biomarkers for major depressive disorder classification based on liquid chromatography-mass spectrometry. J Proteome Res.

[18] Moaddel R, Luckenbaugh DA, Xie Y (2015). D-serine plasma concentration is a potential biomarker of (R,S)-ketamine antidepressant response in subjects with treatment-resistant depression. Psychopharmacology (Berl).

[19] Setoyama D, Kato TA, Hashimoto R (2016). Plasma metabolites predict severity of depression and suicidal ideation in psychiatric patients-a multicenter pilot analysis. PLoS One.

[20] Zheng P, Chen JJ, Zhou CJ (2016). Identification of sex-specific urinary biomarkers for major depressive disorder by combined application of NMR- and GC-MS-based metabonomics. Transl Psychiatry.

[21] Rotroff DM, Corum DG, Motsinger-Reif A (2016). Metabolomic signatures of drug response phenotypes for ketamine and esketamine in subjects with refractory major depressive disorder: new mechanistic insights for rapid acting antidepressants. Transl Psychiatry.

[22] Liu Y, Yieh L, Yang T (2016). Metabolomic biosignature differentiates melancholic depressive patients from healthy controls. BMC Genomics.

[23] Ali-Sisto T, Tolmunen T, Toffol E (2016). Purine metabolism is dysregulated in patients with major depressive disorder. Psychoneuroendocrinology.

[24] Zheng P, Fang Z, Xu XJ (2016). Metabolite signature for diagnosing major depressive disorder in peripheral blood mononuclear cells. J Affect Disord.

[25] Chen JJ, Zhou CJ, Zheng P (2017). Differential urinary metabolites related with the severity of major depressive disorder. Behav Brain Res.

[26] Kageyama Y, Kasahara T, Nakamura T (2018). Plasma nervonic acid is a potential biomarker for major depressive disorder: a pilot study. Int J Neuropsychopharmacol.

[27] Zheng H, Zheng P, Zhao L (2017). Predictive diagnosis of major depression using NMR-based metabolomics and least-squares support vector machine. Clin Chim Acta.

[28] Chen JJ, Bai SJ, Li WW (2018). Urinary biomarker panel for diagnosing patients with depression and anxiety disorders. Transl Psychiatry.

[29] Moaddel R, Shardell M, Khadeer M (2018). Plasma metabolomic profiling of a ketamine and placebo crossover trial of major depressive disorder and healthy control subjects. Psychopharmacology (Berl).

[30] Pan JX, Xia JJ, Deng FL (2018). Diagnosis of major depressive disorder based on changes in multiple plasma neurotransmitters: a targeted metabolomics study. Transl Psychiatry.

[31] Kawamura N, Shinoda K, Sato H (2018). Plasma metabolome analysis of patients with major depressive disorder. Psychiatry Clin Neurosci.

[32] Liu D, Ray B, Neavin DR (2018). Beta-defensin 1, aryl hydrocarbon receptor and plasma kynurenine in major depressive disorder: metabolomics-informed genomics. Transl Psychiatry.

[33] Ali-Sisto T, Tolmunen T, Viinamäki H (2018). Global arginine bioavailability ratio is decreased in patients with major depressive disorder. J Affect Disord.

[34] Liu LY, Zhang HJ, Luo LY (2018). Blood and urinary metabolomic evidence validating traditional Chinese medicine diagnostic classification of major depressive disorder. Chin Med.

[35] Gui SW, Liu YY, Zhong XG (2018). Plasma disturbance of phospholipid metabolism in major depressive disorder by integration of proteomics and metabolomics. Neuropsychiatr Dis Treat.

[36] Czysz AH, South C, Gadad BS, Arning E, Soyombo A, Bottiglieri T, Trivedi MH (2019). Can targeted metabolomics predict depression recovery? Results from the CO-MED trial. Transl Psychiatry.

[37] Bhattacharyya S, Ahmed AT, Arnold M (2019). Metabolomic signature of exposure and response to citalopram/escitalopram in depressed outpatients. Transl Psychiatry.

[38] Bhattacharyya S, Dunlop BW, Mahmoudiandehkordi S (2019). Pilot study of metabolomic clusters as state markers of major depression and outcomes to CBT treatment. Front Neurosci.

[39] Chen JJ, Xie J, Li WW, Bai SJ, Wang W, Zheng P, Xie P (2019). Age-specific urinary metabolite signatures and functions in patients with major depressive disorder. Aging (Albany NY).

[40] Ahmed AT, MahmoudianDehkordi S, Bhattacharyya S (2020). Acylcarnitine metabolomic profiles inform clinically-defined major depressive phenotypes. J Affect Disord.

[41] Brunoni AR, Salum GA, Hoffmann MS (2020). Prospective associations between hsCRP and GlycA inflammatory biomarkers and depression: The Brazilian longitudinal study of adult health ( ELSA-Brasil). J Affect Disord.

[42] Han SY, Tomasik J, Rustogi N (2020). Diagnostic prediction model development using data from dried blood spot proteomics and a digital mental health assessment to identify major depressive disorder among individuals presenting with low mood. Brain Behav Immun.

[43] Erabi H, Okada G, Shibasaki C (2020). Kynurenic acid is a potential overlapped biomarker between diagnosis and treatment response for depression from metabolome analysis. Sci Rep.

[44] Zhao S, Chi A, Yan J, Yao C (2020). Feature of heart rate variability and metabolic mechanism in female college students with depression. Biomed Res Int.

[45] Shen D, Zhao H, Gao S (2021). Clinical serum metabolomics study on fluoxetine hydrochloride for depression. Neurosci Lett.

[46] Du Y, Wei J, Yang X (2021). Plasma metabolites were associated with spatial working memory in major depressive disorder. Medicine (Baltimore).

[47] Gamradt S, Hasselmann H, Taenzer A (2021). Reduced mitochondrial respiration in T cells of patients with major depressive disorder. iScience.

[48] Homorogan C, Nitusca D, Enatescu V, Schubart P, Moraru C, Socaciu C, Marian C (2021). Untargeted plasma metabolomic profiling in patients with major depressive disorder using ultra‐high performance liquid chromatography coupled with mass spectrometry. Metabolites.

[49] Tateishi H, Setoyama D, Kang D (2021). The changes in kynurenine metabolites induced by rTMS in treatment-resistant depression: a pilot study. J Psychiatr Res.

[50] Caspani G, Turecki G, Lam RW (2021). Metabolomic signatures associated with depression and predictors of antidepressant response in humans: a CAN-BIND-1 report. Commun Biol.

[51] Hung CI, Lin G, Chiang MH, Chiu CY (2021). Metabolomics-based discrimination of patients with remitted depression from healthy controls using 1H-NMR spectroscopy. Sci Rep.

[52] Bai S, Xie J, Bai H, Tian T, Zou T, Chen JJ (2021). Gut microbiota-derived inflammation-related serum metabolites as potential biomarkers for major depressive disorder. J Inflamm Res.

[53] Kageyama Y, Deguchi Y, Hattori K, Yoshida S, Goto YI, Inoue K, Kato T (2021). Nervonic acid level in cerebrospinal fluid is a candidate biomarker for depressive and manic symptoms: a pilot study. Brain Behav.

[54] Mocking RJ, Naviaux JC, Li K (2021). Metabolic features of recurrent major depressive disorder in remission, and the risk of future recurrence. Transl Psychiatry.

[55] Brydges CR, Fiehn O, Mayberg HS (2021). Indoxyl sulfate, a gut microbiome-derived uremic toxin, is associated with psychic anxiety and its functional magnetic resonance imaging-based neurologic signature. Sci Rep.

[56] Ciocan D, Cassard AM, Becquemont L (2021). Blood microbiota and metabolomic signature of major depression before and after antidepressant treatment: a prospective case-control study. J Psychiatry Neurosci.

[57] Kurokawa S, Tomizawa Y, Miyaho K (2021). Fecal microbial and metabolomic change during treatment course for depression: an observational study. J Psychiatr Res.

[58] Hu Y, Wang Y, Chen C, Yang W, Zhu W, Wang Y, Liu P (2021). A randomized, placebo-controlled, double-blind study on the effects of SZL on patients with mild to moderate depressive disorder with comparison to fluoxetine. J Ethnopharmacol.

[59] Joyce JB, Grant CW, Liu D (2021). Multi-omics driven predictions of response to acute phase combination antidepressant therapy: a machine learning approach with cross-trial replication. Transl Psychiatry.

[60] de Kluiver H, Jansen R, Milaneschi Y, Bot M, Giltay EJ, Schoevers R, Penninx BW (2021). Metabolomic profiles discriminating anxiety from depression. Acta Psychiatr Scand.

[61] Dhakshinamoorthy S, Dinh NT, Skolnick J, Styczynski MP (2015). Metabolomics identifies the intersection of phosphoethanolamine with menaquinone-triggered apoptosis in an in vitro model of leukemia. Mol Biosyst.

[62] Mello D, Kunzler D, Farah M (2007). A cafeína e seu efeito ergogênico. Br JSports Nutrition.

[63] Shaw K, Turner J, Del Mar C (2002). Are tryptophan and 5-hydroxytryptophan effective treatments for depression? A meta-analysis. Aust N Z J Psychiatry.

[64] MacDonald K, Krishnan A, Cervenka E, Hu G, Guadagno E, Trakadis Y (2019). Biomarkers for major depressive and bipolar disorders using metabolomics: a systematic review. Am J Med Genet B Neuropsychiatr Genet.

[65] Guest PC, Guest FL, Martins-de Souza D (2016). Making sense of blood-based proteomics and metabolomics in psychiatric research. Int J Neuropsychopharmacol.

[66] Zacharias HU, Hertel J, Johar H (2021). A metabolome-wide association study in the general population reveals decreased levels of serum laurylcarnitine in people with depression. Mol Psychiatry.

[67] Kendall KM, Van Assche E, Andlauer TF, Choi KW, Luykx JJ, Schulte EC, Lu Y (2021). The genetic basis of major depression. Psychol Med.

